# Integrated Multidimensional Analysis Is Required for Accurate Prognostic Biomarkers in Colorectal Cancer

**DOI:** 10.1371/journal.pone.0101065

**Published:** 2014-07-02

**Authors:** Marisa Mariani, Shiquan He, Mark McHugh, Mirko Andreoli, Deep Pandya, Steven Sieber, Zheyang Wu, Paul Fiedler, Shohreh Shahabi, Cristiano Ferlini

**Affiliations:** 1 Danbury Hospital Research Institute, Danbury, Connecticut, United States of America; 2 Worcester Polytechnic Institute, Department of Mathematical Sciences, Worcester, Massachusetts, United States of America; University General Hospital of Heraklion and Laboratory of Tumor Cell Biology, School of Medicine, University of Crete, Greece

## Abstract

CRC cancer is one of the deadliest diseases in Western countries. In order to develop prognostic biomarkers for CRC (colorectal cancer) aggressiveness, we analyzed retrospectively 267 CRC patients via a novel, multidimensional biomarker platform. Using nanofluidic technology for qPCR analysis and quantitative fluorescent immunohistochemistry for protein analysis, we assessed 33 microRNAs, 124 mRNAs and 9 protein antigens. Analysis was conducted in each single dimension (microRNA, gene or protein) using both the multivariate Cox model and Kaplan-Meier method. Thereafter, we simplified the censored survival data into binary response data (aggressive vs. non aggressive cancer). Subsequently, we integrated the data into a diagnostic score using sliced inverse regression for sufficient dimension reduction. Accuracy was assessed using area under the receiver operating characteristic curve (AUC). Single dimension analysis led to the discovery of individual factors that were significant predictors of outcome. These included seven specific microRNAs, four genes, and one protein. When these factors were quantified individually as predictors of aggressive disease, the highest demonstrable area under the curve (AUC) was 0.68. By contrast, when all results from single dimensions were combined into integrated biomarkers, AUCs were dramatically increased with values approaching and even exceeding 0.9. Single dimension analysis generates statistically significant predictors, but their predictive strengths are suboptimal for clinical utility. A novel, multidimensional integrated approach overcomes these deficiencies. Newly derived integrated biomarkers have the potential to meaningfully guide the selection of therapeutic strategies for individual patients while elucidating molecular mechanisms driving disease progression.

## Introduction

CRC is one of the deadliest diseases worldwide. Caucasian patients with local, regional, or metastatic disease exhibit a 5-year survival rate of 66%, 44%, and 4%, respectively [1]. Disease stage at the time of surgery is well established as the most important prognostic factor in CRC. In the last two decades, median overall survival has increased significantly with the introduction of new cytotoxic agents and biologic therapies. The response to such treatments depends on molecular determinants whose elucidation has been the focus of intense and productive research efforts. We now know, for example, that cancers harboring activating KRAS mutations do not respond to anti-EGFR therapy [2]. However, the goal of optimizing treatment protocols based on the unique molecular characteristics of an individual's tumor still remains elusive. Development of novel biomarkers that can reliably identify patients at high risk for disease progression and death would be especially useful in determining the clinical circumstances where adjuvant chemotherapy is warranted. Whereas the use of the antimetabolite 5-Fluorouracil (5FU) is standard therapy for patients with stage III CRC, its potential benefits compared to risks in stage II CRC patients is a matter of controversy and debate [3]. In the absence of a robust clinical predictor of disease outcome, the decision to treat or not to treat stage II patients with 5FU cannot rest on objective and firm criteria. Previously identified predictive biomarkers which had shown great promise in this arena including telomerase, transforming growth factors (TGFα and TGFβ), epidermal growth factors (erbB2 and erbB3) and mucin (MUC1 and MUC2) have disappointed in studies of clinical utility [4].

The traditional approach to biomarker development relies on single dimensional (microRNA, gene or protein) analysis in an attempt to link a single molecular entity to tumor behavior. This method seems to have reached a zenith that is suboptimal for clinical decision-making. Previous multidimensional approaches have demonstrated that through the combination of biomarkers coming from different dimensions a better knowledge of the biology of CRC can be achieved [5], [6], [7]. In an attempt to provide more personalized options, we developed a novel method that further advances the integration and incorporates multiple molecular entities from all three molecular dimensions (microRNA, genes and protein) simultaneously to generate accurate predictors of outcome in patients with CRC. Our results clearly demonstrate the superiority of this novel, multidimensional approach as compared with the traditional tools of single dimension analysis. We are hopeful that newly discovered multidimensional biomarkers will provide a basis for successful triage and stratification of patients in prospective clinical trials while simultaneously revealing molecular agents and pathways playing prominent sinister roles in CRC disease progression.

## Materials and Methods

### Gene and micro-RNA expression assessed with nanofluidic technology

A clinical cohort of 267 colon cancer patients was analyzed in this retrospective study. After approval of the Danbury Hospital Internal Review Board (DHIRB) and collection of the relevant clinical information, FFPE samples were obtained from colon cancer cases that had been preserved between 2000 and 2008. According to the protocol of the study (DH-17/12) including full de-identification of patient information, DHIRB waived the need of informed consent. FFPE samples were cut to 10 µm thickness and two tissue slices were put into a 1.5 ml tube. To each tube, one milliliter of xylene was added for deparaffinization followed by mixing twice with a high speed vortex for 3 min at room temperature. Total RNA was then automatically extracted with the QIAcube using the miRNeasy FFPE kit (Qiagen, Valencia, CA) following manufacturer's protocol. The RNA from SW837 cells was automatically extracted with the QIAcube using the miRNeasy kit (Qiagen, Valencia, CA) following manufacturer's protocol. RNA quantity and the quality were assessed by Agilent 2100 Bioanalyzer (Agilent Technologies, Santa Clara, CA). Analysis was carried out using the 48.48 dynamic array (Fluidigm Corporation, CA, USA) and a Biomark platform following the manufacturer's protocol as previously described [8], [9].

### Quantitative fluorescent immunohistochemistry

Quantitative fluorescent immunohistochemistry was performed for protein analysis. Tissue specimens were prepared in a Tissue Micro Array (TMA) format: representative tumor areas were obtained from Formalin Fixed Paraffin Embedded (FFPE) specimens of the primary tumor, and up to three representative replicate 3-mm cores from multiple tumor blocks were taken after review and marking of the hematoxylin and eosin stained slides by board-certified pathologists (SS and PF). In total, 630 cores were taken and distributed over 16 slides from 267 patients. FFPE tissues used as controls of the reaction included normal colon, kidney, liver, brain, breast, lymph nodes, thyroid, skin, tonsil, skeletal muscle and bladder along with breast cancer and non-small cell lung cancer.

TMA slides were deparaffinized in xylene and then rehydrated in sequentially diluted ethanol solutions. Antigen retrieval was conducted by heating the slides in a steamer for 30 minutes in a solution of Tris-EDTA pH 8.0. Endogenous peroxidase activity was blocked by treating the slides in Peroxidazed reagent (Biocare Medical, Concord, CA) for 5 minutes. Non-specific binding was reduced by incubation with Background Sniper (Biocare Medical, Concord, CA) for 10 minutes. Slides were incubated with the primary target antibodies and epithelial and stromal cell mask antibodies diluted in Da Vinci Green antibody diluent (Biocare Medical, Concord, CA) for 1 hour at room temperature. Details of all antibodies used are in Table S1. Cyanine 5 (Cy5) directly conjugated to tyramide (Perkin-Elmer, Boston, MA) at 1∶50 dilution was used as the fluorescent detection for all the target antigens.

### Statistical Analysis

For single dimension analysis, overall survival was calculated from the date of diagnosis to the date of death or date last seen. Medians and life tables were computed using the product-limit estimate by the Kaplan and Meier method, and the Log Rank test was employed only to assess the statistical significance. Multivariate analysis assessed the clinical role of each factor matched with other clinical variables (age, stage, grading, type of tumor, and gender), following Cox proportional hazards model. To simplify the issue of censoring, we remove the patients who were censored within 3 years and transformed the survival data into binary response, either aggressive or non-aggressive. For each factor proven to be significant in multivariate analysis (p-value <0.05), the area under the curve (AUC) in the receiver operating characteristic (ROC) curve was utilized to assess discriminatory power.

For multidimensional analysis, the dataset was randomly divided into training and testing subsets, with 125 cases in each subset. Multiple biomarkers were combined to yield a diagnostic score that was used as a predictor of outcome. To generate the score, we first used sliced inverse regression [10], [11] to do the sufficient dimension reduction whereby no information about the conditional distribution of outcome was lost during the dimension reduction. Next, a scalar diagnostic score was computed from the lower dimensional data generated in the first step by likelihood ratio statistic which has been proven to be optimal among all possible functions of multiple markers for binary disease outcomes [12]. This approach enabled utilization of information from multiple markers simultaneously without the need to make assumptions concerning the distributions of the markers. Cox and Kaplan-Meier models were employed to evaluate the statistical significance of multidimensional biomarkers in multivariate analysis as described above.

## Results

### Expression Analysis of microRNA

**Figure 1:**
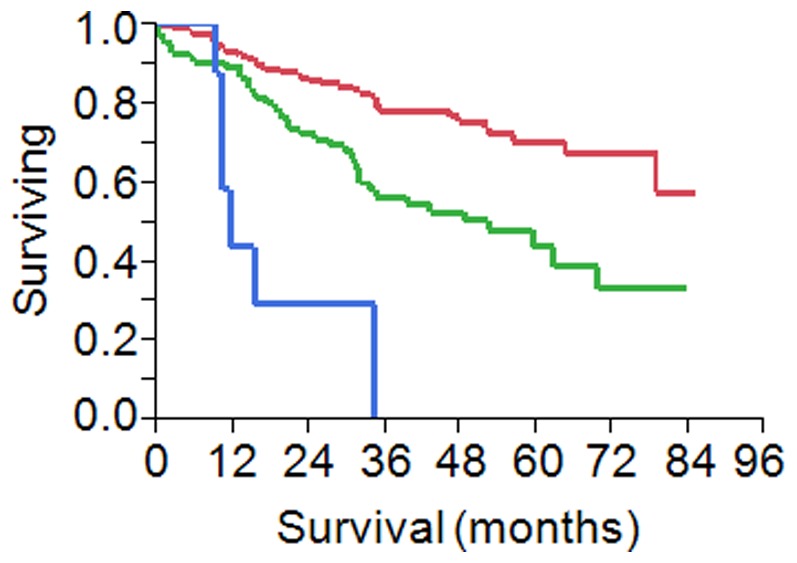
Kaplan-Meier analysis according to stage of the clinical group of 267 patients enrolled in this retrospective analysis. In red stage I–II patients (n = 176), in green stage III patients (n = 82) and in blue stage IV (n = 8).

**Figure 2:**
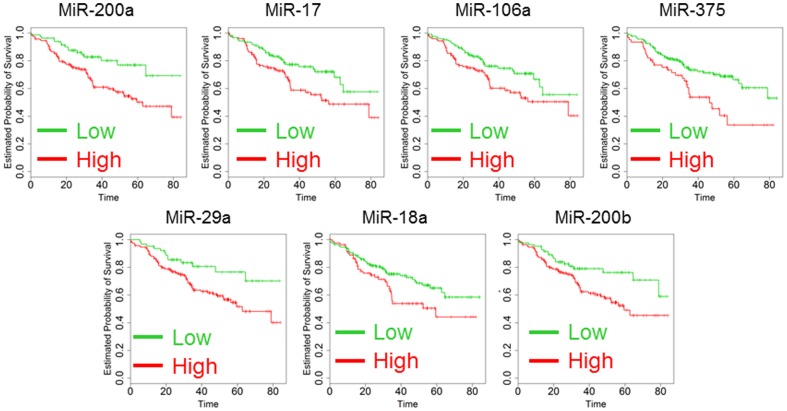
Kaplan-Maier analysis of 267 patients according to the analysis of Mir-200a, Mir-17, Mir-106a, MiR-375, Mir-29a, MiR-18a and Mir-200b expression. Kaplan-Maier analysis was performed dividing the patients as high (green) and low (red) setting. Survival time scale is in months. All the differences were significant and p-values are reported in Table 2 (Log-rank test).

The main clinical parameters of the 267 CRC patients enrolled in this retrospective analysis are illustrated in Table 1. All the specimens were collected at the first surgery before any treatment. As anticipated the most important clinical factor to predict outcome was the stage of the disease. For patients at stage IV the progression was fast with a median survival rate of 11 months, while for patients at earlier stages the outcome was better (Fig. 1). All the patients were then treated with the best available care and this study will focus on pure prognostic predictors and not to predictors of response to specific treatments. As a first step, we screened a series of 33 microRNAs to identify potential predictors of outcome in multivariate analysis including age and stage in the Cox model. MicroRNAs were chosen according to the number of citations in Pubmed using as keywords the terms “colorectal cancer” and “microRNA”. Ten microRNAs (MiR-532-3p, Mir-200a, Mir-17, Mir-106a, MiR-193a-5p, MiR-145, MiR-375, Mir-29a, MiR-18a and Mir-200b) were statistically significant with values of range risk ratio (RR) less than 1 for each, meaning that high expression was related to a good outcome (Table 2). To further support the results of Cox analysis, data were also assessed using the Kaplan-Meier method. Five quintile cutoffs (25, 33, 50, 67 and 75) were used to stratify patients for high and low expression of each microRNA and log-rank test served to detect if differences in the outcome were significant. The quintile cutoff providing the lowest p-value at the log-rank test was used as discriminator (Table 2). Seven microRNAs (Mir-200a, Mir-17, Mir-106a, MiR-375, Mir-29a, MiR-18a and Mir-200b) were confirmed to be significant using the Kaplan-Meier method and the corresponding plots are shown in Fig. 2.

**Table 1:** Distribution of clinico-pathological characteristics of patients.

Characteristics	All Cases (%)	Training Set (%)	Testing Set (%)	p-value*
Number	267	125	125	0.9469
Age, yrs Median (range)	70 (22–96)	72 (22–96)	70 (25–95)	
AJCC Stage				
I–II	176 (65.9)	82 (65.6)	80 (64.0)	0.8947
III–IV	91 (34.1)	43 (34.4)	45 (36.0)	
Gender				
Male	122 (45.6)	55 (44.0)	59 (47.2)	0.7033
Female	145 (54.3)	70 (56.0)	66 (52.8)	
Histotype				
Adenocarcinoma	243 (91.0)	114 (91.2)	115 (92.0)	0.9114
Mucinous	24 (9.0)	11 (8.8)	10 (8.0)	
Grade				
G1-2	212 (78.2)	99 (79.2)	101 (80.8)	0.6091
G3	31 (20.9)	18 (14.4)	13 (10.4)	
N/A	24 (3.8)	8 (6.4)	11 (8.8)	

*testing the difference between training and testing set.

**Table 2:** Results of microRNA expression analysis in the clinical setting of 267 patients with multivariate Cox and Kaplan-Meier method.

	P-value from KM	P-value from Cox	Range risk ratio	Range risk ratio lower limit	Range risk ratio upper limit
MiR-532-3p	0.064771	0.008339	0.094842	0.01648	0.545822
Mir-200a	0.0029	0.010557	0.077561	0.010928	0.550463
Mir-17	0.019042	0.011139	0.079489	0.011251	0.561619
Mir-106a	0.020048	0.015244	0.099816	0.015518	0.642047
MiR-193a-5p	0.071498	0.016423	0.108225	0.0176	0.665506
MiR-145	0.107096	0.019985	0.193244	0.048386	0.771785
MiR-375	0.000434	0.020366	0.21165	0.056987	0.786067
Mir-29a	0.001772	0.024357	0.135351	0.023734	0.771891
MiR-18a	0.020165	0.027317	0.224383	0.059514	0.845988
Mir-200b	0.02183	0.047943	0.144104	0.021133	0.98261
Mir-200c	0.015671	0.053802	0.193341	0.03639	1.027235
Mir-429	0.009123	0.057033	0.180053	0.030799	1.052607
Let-7c	0.122339	0.064338	0.218802	0.043733	1.094688
Mir-126	0.013457	0.07168	0.198886	0.034305	1.15305
Mir-92a	0.015478	0.088624	0.311977	0.081623	1.192433
MiR-128	0.074133	0.091335	0.322118	0.086476	1.199867
MiR-18b	0.057048	0.093705	0.350199	0.102678	1.194406
Mir-141	0.030167	0.100176	0.252985	0.049144	1.302326
Mir-27a	0.03627	0.103385	0.259099	0.051005	1.316174
Let-7g	0.11726	0.104305	0.295949	0.068104	1.286059
Mir-221	0.063595	0.119559	0.322513	0.077581	1.340726
Mir-21	0.103007	0.13283	0.341332	0.084031	1.386486
MiR-320	0.005064	0.155964	0.299032	0.056422	1.584841
MiR-642	0.039954	0.185248	0.280021	0.042591	1.841022
Mir-34a	0.042544	0.237098	0.395142	0.084772	1.84185
Let-7e	0.03418	0.328038	0.514886	0.136147	1.947215
Mir-20a	0.321134	0.331775	0.509387	0.130451	1.989068
MiR-328	0.02598	0.383902	0.551185	0.144202	2.106798
Mir-31	0.343731	0.507135	0.570242	0.108455	2.998244
Mir-183	0.31913	0.572282	0.5731	0.083001	3.95708
MiR-30c	0.291601	0.882707	0.893081	0.198839	4.011246
MiR-125b	0.12562	0.941408	0.947918	0.227684	3.946476
Mir-203	0.033555	0.954696	0.959546	0.230859	3.988278

### Expression Analysis of genes

**Figure 3:**
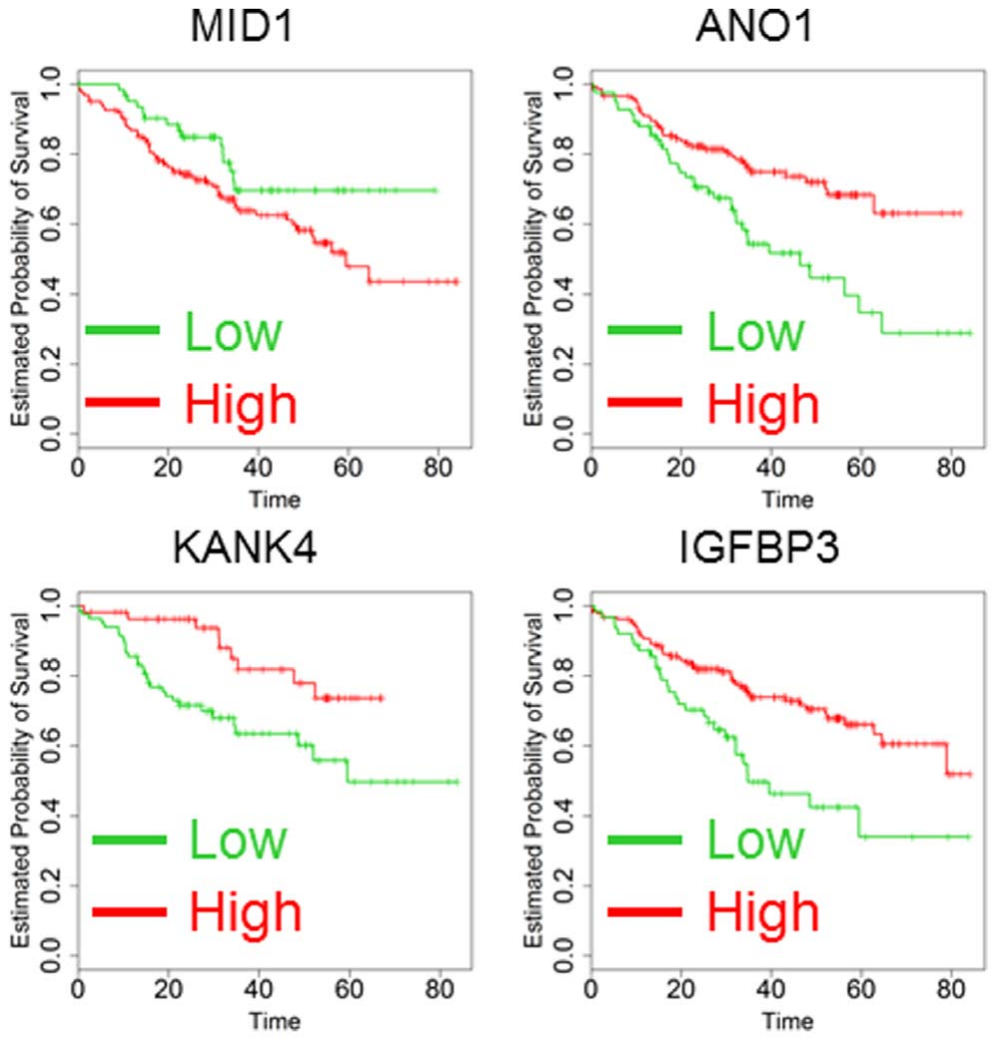
Kaplan-Maier analysis of 267 patients according to the analysis of MID1, ANO1, KANK4 and IGFBP3 expression. Kaplan-Maier analysis was performed dividing the patients as high (green) and low (red) setting. Survival time scale is in months. All the differences were significant and p-values are reported in Table 3 (Log-rank test).

Nanofluidic technology offers the advantage of allowing analysis of microRNAs and their target genes (targetome) in the same RNA sample due to the low volume of each individual qPCR analysis. To perform this analysis, we employed multiple software applications (www.miRbase.org)[13] to prepare a list of genes that might be targetable by the 33 microRNAs investigated in this study. The list was prioritized according to a functional network obtained with the DAVID software (http://david.abcc.ncifcrf.gov) [14] in order to enrich the pool with actionable targets and master regulators of gene expression and apoptosis. After an initial analysis of 180 candidates, we focused on 79 genes whose expression was detectable in a large number of CRC cancer patients. Six genes (MID1, INHBA, OSBPL3, BGN, DICER1 and FAP) were predictors in univariate analysis (data not shown), but only MID1 remained significant after multivariate correction and Kaplan-Meier analysis (Table 3 and Fig. 3). As a second measure to possibly increase the number of candidate genes, we analyzed the public dataset GSE14333 reporting transcriptome analysis of 290 CRC cancer patients [15]. For each individual gene, data were analyzed and computed in a multivariate Cox model as described above (Table 4). Predictive capability was confirmed by Kaplan-Maier analysis using a multiple procedure of quintile selection for the cutoff as described above. The 45 genes with the lowest p-values in multivariate analysis were assessed in our platform of nanofluidic gene expression. Only 3 out of 45 (7%) genes were confirmed as predictors of outcome in both GSE14333 and our clinical setting in multivariate Cox regression and Kaplan-Meier analysis (ANO1, KANK4 and IGFBP3, Table 4 and Fig. 3).

**Table 3:** Results of gene expression analysis in the clinical setting of 267 patients with multivariate Cox and Kaplan-Meier method.

	P-value from KM	P-value from Cox	Range risk ratio	Range risk ratio lower limit	Range risk ratio upper limit
ANO1	0.000455	0.001999	251.7049	7.554459	8386.487
MID1	0.022369	0.00219	0.061488	0.010323	0.366259
KANK4	0.00051	0.014574	29.03691	1.946079	433.2518
IGFBP3	0.000968	0.021595	14.17555	1.476178	136.1259
KLF6	0.239788	0.02687	0.130799	0.021595	0.792224
GLI3	0.114457	0.038389	0.115389	0.014944	0.890962
SMCR7L	0.037868	0.04224	9.413194	1.081742	81.91252
NEK6	0.066407	0.050178	133.963	0.996207	18014.43
NAT2	0.356728	0.065965	7.387218	0.876405	62.26683
PBX3	0.140048	0.072855	0.076392	0.004598	1.26913
ANGPT2	0.000343	0.073683	21.83904	0.744166	640.9105
AIMP2	0.431665	0.07928	16.0743	0.722844	357.4535
OSBPL3	8.41E-05	0.08083	4.974909	0.821414	30.13061
ATXN1	0.020848	0.102364	8.834232	0.646996	120.6247
TUBB3	0.019182	0.106898	3.208144	0.777738	13.2335
TPBG	0.009543	0.109652	3.902565	0.735954	20.69425
IGF1R	0.134023	0.110324	9.739804	0.595836	159.2113
EZR	0.016156	0.117108	17.77504	0.485969	650.1487
SAV1	0.011384	0.118547	14.03151	0.509015	386.7929
AR	0.348534	0.121365	0.161848	0.01616	1.621013
RAI14	0.00433	0.131537	3.122865	0.710897	13.71828
CXCR4	0.05891	0.140109	0.269459	0.047199	1.53833
UST	0.013135	0.140332	7.706586	0.510569	116.324
INHBA	0.002297	0.145151	3.199909	0.669214	15.30066
CCL5	0.07661	0.146705	0.218204	0.027924	1.705063
STC1	0.047976	0.154726	11.71039	0.395198	346.9982
CALU	0.540614	0.173061	0.215761	0.02376	1.959315
CD109	0.159253	0.178425	5.255192	0.46883	58.90633
DICER1	0.166057	0.19096	0.255369	0.033012	1.975454
HIF1A	0.779411	0.208162	0.362001	0.074392	1.76154
MKI67	0.007381	0.224242	3.396451	0.472755	24.40137
VAV3	0.024025	0.224528	0.47273	0.141103	1.583763
TFAM	0.482992	0.240308	10.34319	0.209424	510.8378
BGN	0.013917	0.24456	2.629649	0.516015	13.40088
UGCG	0.133881	0.245772	18.61169	0.133532	2594.106
PLK2	0.121792	0.271772	7.080799	0.215788	232.3469
CAV2	0.095172	0.276778	2.660236	0.456206	15.51242
BBX	0.374652	0.279638	12.47811	0.128517	1211.536
SNAI1	0.183131	0.282896	0.363617	0.057377	2.304384
MCL1	0.231297	0.283847	0.295541	0.031804	2.746347
COX7A2L	0.122784	0.285949	0.277405	0.026316	2.924187
CEP170	0.018982	0.299886	4.728032	0.250678	89.17529
CCND1	0.171718	0.300918	3.251198	0.348254	30.35228
COMMD2	0.077355	0.303629	5.395563	0.217408	133.9053
ADAMTS5	0.123317	0.305862	2.314392	0.464361	11.535
ESR2	0.153752	0.311968	2.66113	0.399116	17.74326
KLF12	0.465026	0.319069	2.64606	0.390215	17.943
CCL2	0.441674	0.319525	0.438755	0.086651	2.22162
EPAS1	0.490625	0.331663	0.418505	0.072098	2.429288
RTN2	0.249843	0.334748	2.735875	0.353963	21.14633
CHST13	0.039054	0.336699	2.876949	0.33316	24.84341
PIM1	0.105052	0.367117	3.552454	0.226011	55.83778
PPP2CA	0.19979	0.373204	0.317213	0.025341	3.970806
CLOCK	0.209762	0.374067	0.492709	0.103457	2.346506
FAM84A	0.107645	0.37997	0.496851	0.104249	2.367992
TM4SF1	0.158023	0.392541	3.496384	0.198371	61.62552
CAMK2D	0.703007	0.396634	1.968245	0.411242	9.420213
HIPK1	0.368779	0.397418	0.322743	0.023514	4.429794
KLF5	0.089271	0.408022	0.523601	0.113069	2.42469
PTEN	0.554417	0.413923	0.412612	0.049346	3.450134
MYOF	0.275264	0.417654	5.561907	0.087722	352.6461
PNRC1	0.318134	0.420618	3.398165	0.173127	66.69977
IGF2R	0.096886	0.429115	2.60505	0.242724	27.95882
HGF	0.003672	0.429973	2.099431	0.332818	13.24333
CDKN1A	0.059386	0.430643	0.388964	0.037156	4.071878
TGFB1	0.006336	0.446801	2.421279	0.248095	23.63038
KDR	0.18116	0.449974	1.72842	0.417918	7.14838
EPHA3	0.567195	0.472304	0.55043	0.108045	2.804128
CFTR	0.384598	0.47535	2.360475	0.223261	24.95658
ARNT2	0.06533	0.481856	0.413958	0.035442	4.834986
MITF	0.110527	0.487799	1.829958	0.331946	10.08821
AHNAK2	0.188641	0.495026	1.774388	0.341724	9.213451
KLF7	0.049811	0.506272	1.612536	0.39409	6.598172
JAK2	0.394445	0.516395	0.591344	0.120979	2.890488
SOX2	0.150192	0.541916	0.530877	0.069385	4.061828
MAPRE1	0.357987	0.543075	0.557944	0.085098	3.658149
CDH1	0.03582	0.552333	1.615003	0.332349	7.847868
IGSF5	0.019559	0.555833	1.679289	0.29926	9.423273
ANTXR2	0.546413	0.565429	3.671851	0.043523	309.7803
PLK1	0.416995	0.572943	0.671878	0.168582	2.677758
MECP2	0.462499	0.581138	0.626129	0.118665	3.303723
VAMP2	0.161211	0.58226	0.628316	0.119973	3.290569
COL1A1	0.02623	0.584364	0.529685	0.054348	5.162451
FES	0.382467	0.593556	1.591399	0.288902	8.766134
PTK2	0.060876	0.601408	1.649351	0.25232	10.78138
BCL2	0.338186	0.610589	0.626785	0.103814	3.784251
HOXB5	0.27284	0.62191	1.486459	0.307571	7.183917
CYP39A1	0.486591	0.642143	1.671365	0.191529	14.58506
CDX2	0.002486	0.683233	0.651889	0.083486	5.090169
PDGFRB	0.042645	0.69718	1.42049	0.242459	8.322194
CD59	0.005198	0.700335	1.635662	0.133489	20.04205
EPHB2	0.042245	0.744099	0.655446	0.051878	8.281188
FAM1738	0.112702	0.744395	1.849962	0.045828	74.67826
SHMT2	0.040828	0.745961	0.73874	0.118273	4.614223
SLC12A2	0.434188	0.771848	0.776719	0.140768	4.285722
HOXB7	0.195627	0.788445	0.798834	0.154871	4.120429
PBK	0.445747	0.790426	0.821722	0.193118	3.496454
DUSP10	0.13842	0.794739	1.669082	0.03519	79.16626
CISH	0.090655	0.810002	0.754679	0.076079	7.486208
FAP	0.030751	0.811833	1.191001	0.282441	5.022236
MAFF	0.255153	0.814144	0.748927	0.067236	8.342135
MET	0.13437	0.823224	1.374103	0.084554	22.33087
CHEK1	0.163926	0.825053	0.822722	0.145827	4.641614
ESR1	0.056936	0.846116	1.3123	0.084339	20.41915
CDX1	0.010579	0.848595	1.30181	0.086808	19.52242
ADM	0.262582	0.853341	1.156516	0.247503	5.40409
HECTD2	0.353439	0.855316	0.860183	0.170415	4.341829
PPARGC1B	0.37968	0.855611	1.222648	0.14027	10.65707
LDLR	0.355447	0.858784	1.222084	0.134155	11.13256
HIC2	0.176202	0.862587	0.815525	0.081015	8.209401
CHEK2	0.155676	0.866904	1.172525	0.182286	7.542064
AXL	0.039057	0.882521	1.167514	0.149675	9.107027
SRC	0.503974	0.883268	0.895666	0.205758	3.898842
FGFR1	0.416422	0.892855	0.896111	0.181618	4.421444
CXCL10	0.090745	0.898925	0.868597	0.098801	7.636207
ETS2	0.135175	0.904279	0.80061	0.021349	30.02398
KIF11	0.243986	0.91388	0.918496	0.196749	4.287873
ROCK1	0.302625	0.915522	1.123317	0.131032	9.630014
DROSHA	0.25995	0.924716	1.086434	0.194645	6.064063
JAK1	0.113129	0.930365	0.905077	0.096647	8.47581
ERBB2	0.302875	0.962123	1.048409	0.14901	7.376446
CTNNB1	0.099735	0.963275	1.03343	0.254908	4.18966
GBP1	0.040014	0.969138	1.043144	0.122758	8.864152
SLITRK4	0.238082	0.973046	0.978316	0.274291	3.489367

**Table 4:** Results of gene expression analysis in the public dataset GSE14333 with multivariate Cox and Kaplan-Meier method.

Probe	Gene	P-value from KM	P-value from Cox	Range risk ratio	Range risk ratio lower limit	Range risk ratio upper limit
229357_at	ADAMTS5	6.04E-06	0.000201	34.0864	5.305339	219.0025
235368_at	ADAMTS5	1.32E-06	8.75E-05	42.64678	6.539883	278.101
219935_at	ADAMTS5	8.92E-05	0.000265	22.25267	4.200208	117.8945
1558636_s_at	ADAMTS5	0.001966	0.005793	100.589	3.802689	2660.788
220726_at	AHNAK2	0.26889	0.690171	0.697388	0.118539	4.102851
1558378_a_at	AHNAK2	0.000362	0.00308	27.08616	3.047499	240.7416
212992_at	AHNAK2	1.55E-06	3.46E-05	483.5156	25.93489	9014.392
209971_x_at	AIMP2	0.000178	0.000173	0.056117	0.01248	0.252328
202138_x_at	AIMP2	0.000483	5.44E-05	0.0415	0.00885	0.194598
209972_s_at	AIMP2	0.012259	0.457615	0.415432	0.040911	4.218525
205572_at	ANGPT2	0.007138	0.027838	11.73135	1.307621	105.248
236034_at	ANGPT2	0.003749	0.000137	102.5228	9.496737	1106.794
211148_s_at	ANGPT2	0.000103	0.066738	26.07117	0.798486	851.2436
237261_at	ANGPT2	0.032343	0.153008	12.34905	0.392984	388.0543
1555269_a_at	ANO1	0.005692	0.02366	23.37811	1.52436	358.5346
218804_at	ANO1	0.001514	0.000249	88.27612	8.031295	970.2886
1555536_at	ANTXR2	0.165872	0.448339	1.992017	0.33543	11.82999
225524_at	ANTXR2	0.002183	0.00047	44.01017	5.278853	366.916
228573_at	ANTXR2	0.005047	0.000316	19.32602	3.857263	96.82903
213015_at	BBX	0.003728	0.006287	20.99437	2.364569	186.4033
1557239_at	BBX	0.291266	0.980533	1.036403	0.058639	18.31775
223134_at	BBX	6.95E-05	0.00274	30.21056	3.248415	280.9611
226331_at	BBX	2.81E-05	8.81E-07	359.268	34.41053	3750.987
213016_at	BBX	0.004471	0.000586	102.7534	7.326352	1441.135
223135_s_at	BBX	6.36E-08	1.22E-05	45.64845	8.239986	252.8865
232008_s_at	BBX	0.000531	9.24E-05	102.4825	10.06176	1043.82
1557240_a_at	BBX	0.169963	0.299311	2.43368	0.453789	13.05188
213426_s_at	CAV2	0.188766	0.031867	7.132542	1.18575	42.90378
203324_s_at	CAV2	3.97E-07	4.18E-05	41.99252	7.026959	250.9438
203323_at	CAV2	1.60E-09	2.04E-06	77.00721	12.82191	462.4983
229900_at	CD109	0.041603	0.004916	26.16536	2.689981	254.5096
226545_at	CD109	0.000325	0.00023	20.46414	4.105505	102.0048
200984_s_at	CD59	0.000362	0.000149	55.54448	6.96658	442.8556
200983_x_at	CD59	1.14E-06	3.41E-05	70.30158	9.407096	525.3813
200985_s_at	CD59	9.51E-06	3.67E-05	52.14801	7.975754	340.9602
212463_at	CD59	6.84E-07	1.22E-06	175.8647	21.78629	1419.626
228748_at	CD59	0.004633	0.041237	7.88942	1.085611	57.33449
206430_at	CDX1	6.36E-07	4.45E-05	0.030374	0.005675	0.162561
206387_at	CDX2	0.0014	3.67E-05	0.073955	0.021472	0.254721
231606_at	CDX2	0.000568	0.095068	0.303118	0.074635	1.231068
212746_s_at	CEP170	0.000299	0.009884	23.82942	2.142353	265.055
207719_x_at	CEP170	0.000131	6.49E-05	31.19191	5.766271	168.7287
234702_x_at	CFTR	0.01694	0.27293	0.461545	0.115856	1.838699
215703_at	CFTR	0.037325	1.37E-05	0.053111	0.014147	0.199386
217026_at	CFTR	0.385981	0.252799	0.168605	0.007976	3.563921
215702_s_at	CFTR	0.012329	0.000782	0.02279	0.002509	0.206981
234706_x_at	CFTR	0.371555	0.578887	0.511618	0.047986	5.454732
205043_at	CFTR	0.021692	3.17E-05	0.065349	0.018078	0.236224
239647_at	CHST13	0.000319	3.61E-05	0.07416	0.021584	0.254811
242503_at	CHST13	0.246465	0.263741	0.463753	0.120515	1.784564
223377_x_at	CISH	0.01124	0.030594	0.153875	0.02821	0.839335
223961_s_at	CISH	4.08E-05	5.85E-05	0.033215	0.006312	0.174775
221223_x_at	CISH	0.002772	0.001846	0.036573	0.004558	0.293441
226910_at	COMMD2	0.000633	0.000126	37.40157	5.872115	238.2237
223491_at	COMMD2	3.60E-05	0.019365	10.47272	1.462487	74.99413
221563_at	DUSP10	5.91E-05	0.001099	37.05756	4.233756	324.3604
215501_s_at	DUSP10	1.84E-05	0.000388	26.5064	4.335402	162.0586
209588_at	EPHB2	0.00033	9.89E-05	0.05185	0.011687	0.230028
209589_s_at	EPHB2	0.010517	0.00193	0.082811	0.017148	0.399908
211165_x_at	EPHB2	2.20E-05	0.000149	0.046433	0.0095	0.226942
210651_s_at	EPHB2	0.027564	0.000968	0.060346	0.011386	0.319845
234158_at	EPHB2	0.031839	0.169435	0.412742	0.116806	1.45845
233699_at	EPHB2	0.231256	0.453665	1.688996	0.428775	6.653159
222303_at	ETS2	0.006806	0.005887	0.114387	0.024448	0.535198
201328_at	ETS2	0.000411	8.08E-05	0.081369	0.023375	0.283247
201329_s_at	ETS2	0.000328	8.38E-05	0.065016	0.016656	0.253792
208621_s_at	EZR	0.034737	0.097102	7.073765	0.7013	71.35055
217234_s_at	EZR	0.044295	0.041498	9.910504	1.092409	89.90964
208622_s_at	EZR	0.000622	0.001303	99.00849	6.013656	1630.07
208623_s_at	EZR	4.02E-07	2.11E-05	1207.412	45.89005	31768.2
217230_at	EZR	0.174403	0.140414	3.361179	0.670718	16.84393
238645_at	EZR	0.021088	0.042168	11.38081	1.089858	118.8439
215200_x_at	EZR	0.286994	0.318044	2.823012	0.368122	21.64879
225670_at	FAM173B	2.61E-05	0.000117	0.033282	0.005894	0.187931
225668_at	FAM173B	0.063779	0.343216	0.331004	0.033645	3.256445
234335_s_at	FAM84A	1.84E-05	2.34E-05	0.021597	0.003653	0.127692
229546_at	FAM84A	0.000894	2.59E-05	0.034042	0.007048	0.164431
225667_s_at	FAM84A	0.000701	1.19E-05	0.013224	0.001908	0.091637
234331_s_at	FAM84A	0.000489	3.23E-07	0.010521	0.001834	0.06036
231439_at	FAM84A	0.002881	0.00016	0.043208	0.008456	0.220772
228459_at	FAM84A	0.000124	0.00285	0.104682	0.023768	0.461058
228319_at	FAM84A	0.088904	0.688981	0.609438	0.053918	6.888528
210095_s_at	IGFBP3	0.00038	0.000122	34.81351	5.693815	212.8592
212143_s_at	IGFBP3	3.87E-06	6.58E-05	40.75663	6.598764	251.7294
243027_at	IGSF5	0.000113	0.001432	55.30184	4.691637	651.8607
229125_at	KANK4	8.48E-06	0.000276	12.86927	3.247498	50.99869
217173_s_at	LDLR	0.011169	0.16538	4.576761	0.533656	39.25138
202067_s_at	LDLR	0.002542	0.120351	14.71872	0.494593	438.0182
202068_s_at	LDLR	0.002236	0.000255	51.66079	6.236509	427.9376
217103_at	LDLR	0.340416	0.568331	0.54143	0.065787	4.456002
217183_at	LDLR	0.129907	0.480234	1.708543	0.386148	7.559592
217005_at	LDLR	0.074924	0.228696	2.675823	0.538828	13.28814
205193_at	MAFF	0.000158	0.000102	44.54421	6.562634	302.3461
211864_s_at	MYOF	1.04E-05	0.000138	62.98781	7.483162	530.1855
201798_s_at	MYOF	0.000208	2.47E-05	188.5189	16.51764	2151.602
217518_at	MYOF	0.020004	0.166863	4.972052	0.511631	48.31859
206797_at	NAT2	0.000112	2.13E-05	0.062607	0.017448	0.224656
201939_at	PLK2	0.003791	7.69E-05	14.06108	3.792438	52.13371
209034_at	PNRC1	1.45E-05	4.87E-05	31.43427	5.954339	165.9484
1555282_a_at	PPARGC1B	0.060267	0.352001	0.505394	0.120092	2.126897
1553639_a_at	PPARGC1B	3.01E-06	2.18E-05	0.03799	0.008394	0.171939
1563943_at	PPARGC1B	0.458245	0.985653	1.014717	0.206439	4.98768
202052_s_at	RAI14	5.41E-07	0.000287	2081.501	33.49958	129334.4
204217_s_at	RTN2	0.000905	0.007263	165.478	3.970497	6896.612
222573_s_at	SAV1	0.002144	0.027685	13.52864	1.331125	137.4957
218276_s_at	SAV1	1.68E-06	4.00E-05	39.62951	6.846883	229.3742
234491_s_at	SAV1	0.023935	0.012655	15.70887	1.802495	136.9039
236606_at	SAV1	0.028297	0.161562	149.8623	0.134718	166709.5
204404_at	SLC12A2	3.73E-06	0.000236	0.05294	0.011053	0.253562
225835_at	SLC12A2	0.001286	0.007522	0.111752	0.022405	0.557401
232636_at	SLITRK4	8.73E-06	0.000342	1081.219	23.6349	49462.24
204596_s_at	STC1	0.001429	0.002766	128.3408	5.339502	3084.814
204595_s_at	STC1	9.53E-06	0.000234	26.79181	4.6472	154.4589
230746_s_at	STC1	0.000866	0.000953	17.63863	3.214301	96.79288
204597_x_at	STC1	2.33E-06	0.002335	76.65634	4.689406	1253.079
238443_at	TFAM	0.059819	0.225228	0.371229	0.074838	1.841456
203177_x_at	TFAM	1.71E-05	0.000113	0.022829	0.00335	0.155556
208541_x_at	TFAM	0.002744	0.054875	0.216047	0.045206	1.032524
203176_s_at	TFAM	0.000342	0.003043	0.115476	0.027697	0.481457
238168_at	TM4SF1	0.030768	0.014898	614.9803	3.498935	108090.2
209387_s_at	TM4SF1	2.56E-08	2.02E-06	800.6651	50.79976	12619.44
215034_s_at	TM4SF1	3.95E-07	1.61E-07	1067.398	78.60081	14495.26
209386_at	TM4SF1	5.87E-10	8.06E-08	1440.194	101.0956	20516.8
215033_at	TM4SF1	0.000849	0.024728	76.44376	1.736114	3365.936
203476_at	TPBG	6.74E-05	0.000102	53.32757	7.179862	396.0842
224967_at	UGCG	0.000167	0.000189	25.07686	4.619337	136.1341
204881_s_at	UGCG	0.030888	0.25574	2.664385	0.491631	14.43957
221765_at	UGCG	6.94E-05	0.01459	18.46403	1.778815	191.6559
205138_s_at	UST	0.039899	0.095085	3.788817	0.792891	18.10481
205139_s_at	UST	0.000152	8.32E-05	39.8298	6.355425	249.6155
214792_x_at	VAMP2	0.00019	0.000615	45.85393	5.13567	409.4078
201556_s_at	VAMP2	0.00036	0.000231	51.11359	6.295788	414.9757
201557_at	VAMP2	0.001365	0.001694	1058.693	13.68399	81908.26

### Expression Analysis of proteins

**Figure 4:**
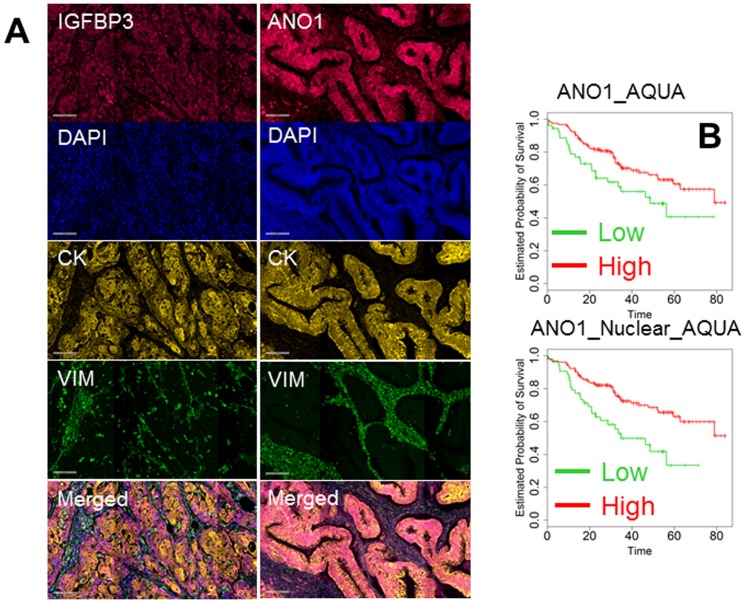
Representative quantitative immunofluorescence for IGFBP3 (left column) and ANO1 (right column). A: From top to bottom the following signals are represented: antigen of interest (red channel), cell nuclei (DAPI), tumor cells (cytokeratin), stromal cells (vimentin) and merged image. B: Kaplan-Maier analysis of 267 patients according to the expression of AQUA scores of ANO1 inside the tumor mask (ANO1_AQUA) and in the nucleus of cancer cells (ANO1_Nuclear_AQUA). Kaplan-Maier analysis was performed dividing the patients as high (green) and low (red) setting. All the differences were significant and p-values are reported in Table 5 (Log-rank test).

To conduct analysis at the protein level, we chose 9 factors (TUBB3, ELAVL1, OSBPL3, IGFBP3, ANO1, HGF, GLI3, PPP2CA and ARNT2). TMAs were prepared from the same paraffin blocks used for gene and microRNA analysis. Triplicate cores of each case were included in the TMAs to capture clonal heterogeneity, and each TMA was analyzed in triplicate by multiplexed, quantitative fluorescent immunohistochemistry. Nuclei were stained with DAPI (blue channel), and stromal and epithelial cells were stained with anti-vimentin (green channel) and anti-cytokeratin (yellow channel), respectively. Antigens of interest were acquired in the red channel, and a representative image of the analysis for IGFBP3 and ANO1 is shown in Fig. 4A. For each protein, expression was quantified with AQUA software which utilizes an unsupervised algorithm to quantify expression in defined subcellular compartments or “masks”. In our study, we selected four masks: tumor (cytokeratin+), stromal (vimentin+), tumor nuclei (DAPI+/cytokeratin+) and tumor cytoplasm (DAPI-/cytokeratin+). For each 3 mm core, at least three electronic subsegments (histospots) were analyzed. Because of replicate analysis, we collected up to 18 AQUA scores for each patient which were then averaged. GLI3, ARNT2 and HGF showed predominantly nuclear staining in some cancer cells, while in others, the staining was predominantly cytoplasmic. To exploit this phenomenon, an index was created by dividing the nuclear over the cytoplasmic expression. A value >1 was typical of a strong nuclear staining, while a value <1 indicated a predominantly cytoplasmic pattern of expression. Expression of all proteins and the index were analyzed with multivariate Cox regression analysis. Only expression of ANO1 (in cancer cells and in the nuclei of cancer cells) was significant in multivariate analysis (Table 5 and Fig. 4B).

**Table 5:** Results of quantitative fluorescent immunohistochemistry quantified with AQUA in the clinical setting of 267 patients with multivariate Cox and Kaplan-Meier method.

	P-value from KM	P-value from Cox	Range risk ratio	Range risk ratio lower limit	Range risk ratio upper limit
ANO1_Nuclear_AQUA	0.019325	0.0163	5.334706	1.360938	20.91138
ANO1_AQUA	0.000898	0.047176	3.933242	1.017225	15.20842
ANO1_Cyto_AQUA	0.000466	0.064055	3.455312	0.930134	12.83598
OSBPL3_Cyto.AQUA	0.123325	0.07897	0.188465	0.029282	1.213009
OSBPL3_Nuclear_AQUA	0.109894	0.080147	0.19641	0.031728	1.21585
ARNT2_Cyto_AQUA	0.167487	0.140698	3.728865	0.647353	21.47889
ANO1_Stromal_AQUA	0.196194	0.168063	2.759558	0.651671	11.68558
ARNT2_Nuclear_AQUA	0.216922	0.190952	3.466272	0.537979	22.33368
HGF_Index	0.072421	0.256612	0.421516	0.094756	1.875095
IGFBP3_Nuclear_AQUA	0.103525	0.280396	2.469812	0.478288	12.75378
IGFBP3_AQUA	0.03215	0.334276	2.071344	0.472373	9.082782
IGFBP3_Cyto_AQUA	0.022362	0.355887	1.91389	0.482418	7.592945
OSBPL3_AQUA	0.253341	0.414725	3.737845	0.157242	88.85318
IGFBP3_Stroma_AQUA	0.256147	0.43221	1.819391	0.408595	8.101375
Gli3_Cytoplasm_AQUA	0.630287	0.440849	1.603306	0.482704	5.3254
HGF_Cyto_AQUA	0.460081	0.504868	0.586884	0.122545	2.810666
Gli3_Index	0.282345	0.566901	0.656363	0.155334	2.773461
ARNT2_Index	0.419295	0.615751	0.562225	0.059327	5.328067
Gli3_Nuclear_AQUA	0.472219	0.621925	1.339116	0.419535	4.274326
ELAVL1_AQUA	0.185824	0.642386	1.911476	0.124104	29.44091
PPP2CA_AQUA	0.431568	0.706374	0.667826	0.081745	5.455859
TUBB3_Aqua	0.142229	0.752119	1.238951	0.327804	4.682667
HGF_AQUA	0.244703	0.75816	0.76795	0.143024	4.12343
HGF_Nuclear_AQUA	0.403127	0.790899	0.785888	0.13239	4.665154
ARNT2_AQUA	0.137421	0.793627	1.212292	0.286559	5.128612
Gli3_AQUA	0.405624	0.813404	0.833907	0.184549	3.768124
TUBB3_Cyto_AQUA	0.253091	0.857207	0.884385	0.23196	3.371855
ELAVL1_Cyto_AQUA	0.375018	0.900491	1.157521	0.116881	11.4634
ELAVL1_Index	0.296773	0.915789	0.90653	0.147036	5.58907
TUBB3_Nuclear_AQUA	0.443265	0.919185	0.947465	0.334061	2.68721
ELAVL1_Nuclear_AQUA	0.037163	0.958367	1.07847	0.063246	18.39011

Nuclear and Cyto indicate expression of the antigen in nucleus and cytoplasm, respectively. If no specified expression was assessed inside the cancer cells. Stromal expression refers to vimentin-positive cells. Index was created dividing the nuclear over the cytoplasmic expression.

### Calculation of Predictive Accuracy

We divided the patients into two clinical groups of interest to allow simplification of censored data into a binary response. Those surviving less than three years from diagnosis were labeled as having aggressive disease, while those surviving for greater than three years were considered to have more indolent, non-aggressive disease. Each of the individual factors from the three dimensions above (microRNA, gene or protein) was tested as a predictor of disease aggressiveness using ROC curves with AUC calculation. Although some factors were statistically significant in multivariate analysis, the maximum AUC obtained from any single biomarker in a single dimension was only 0.68 (ADAMTS5). Utilizing such a weak predictor for patient care would be unacceptable as it is inaccurate (either falsely positive or falsely negative) in approximately one third of the cases.

### Generation of multidimensional biomarkers

**Figure 5:**
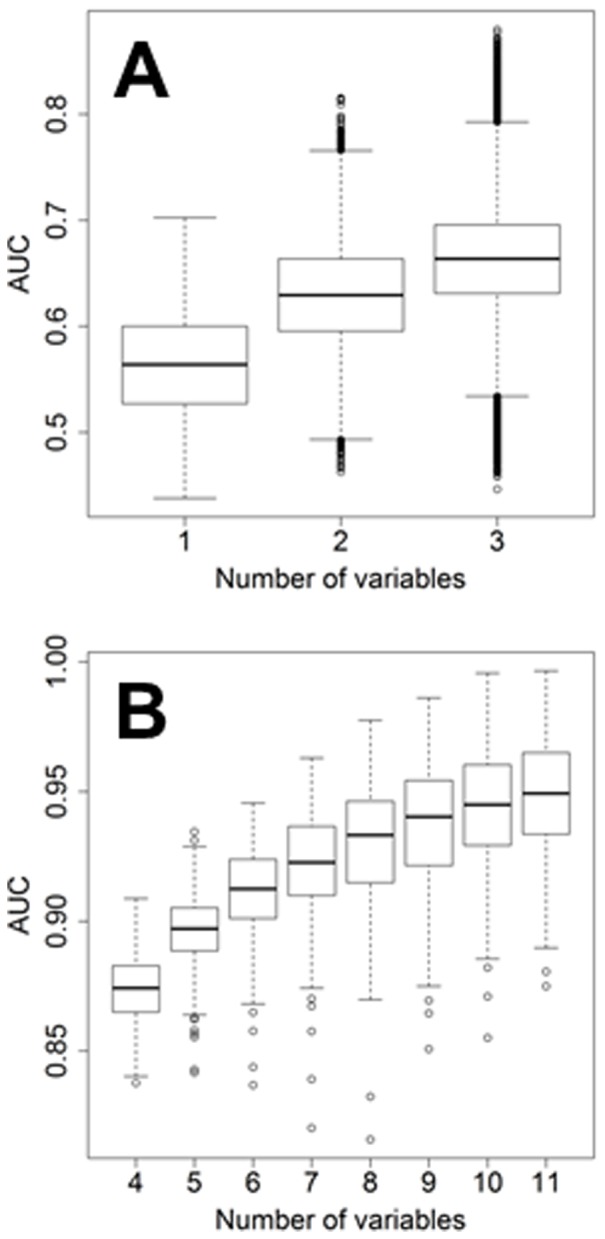
Box-whisker plot representing the values of AUC in the training set. In the boxplot, from bottom to top, they are Q1-1.5*IQR, Q1, median, Q3, and Q3+1.5*Q3 where Q1 is first quartile (25^th^ percentile), Q3 is the third quartile (75^th^ percentile), and IQR is the interquartile (namely, Q3-Q1). In A the analysis is made with a single variable, with all the possible combination of two (n = 17,578) and three variables (n = 1,089,836). In B the analysis is performed by adding one new variable (gene, microRNA or protein) to the previous top combinations.

**Figure 6:**
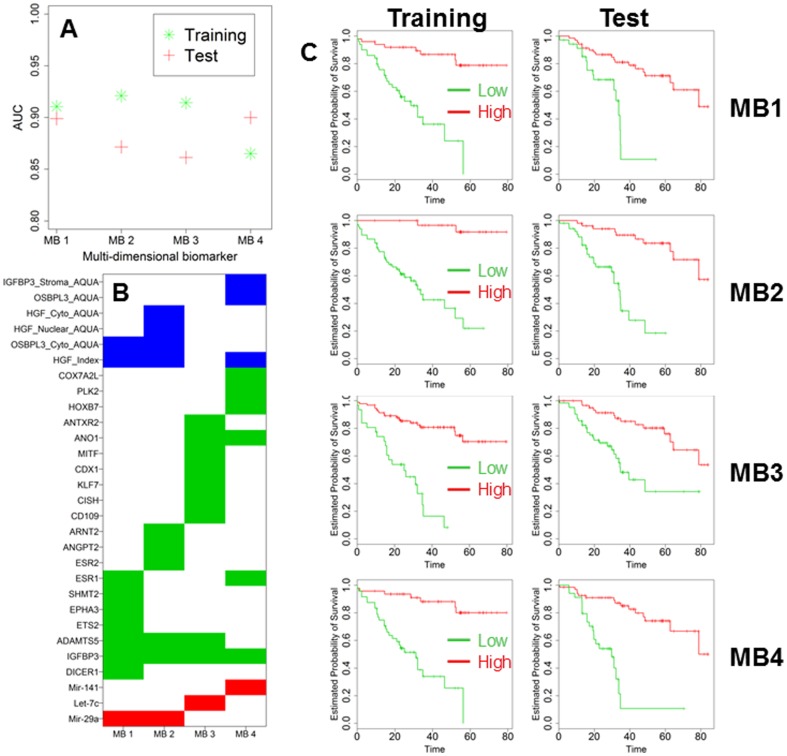
Bar Chart reporting AUC of the top MB obtained in the training and testing set (A). Graphical chart of the composition of MB1-4 (B). In blue, green and red protein, genes and microRNA are reported. Kaplan-Maier analysis of the training and testing set according to the expression of the top 4 MB (C). All the differences were highly significant (Log-rank test) and are reported in Table 7 and 8 for training and testing set, respectively.

We speculated that, by combining the information from different dimensions (microRNA, gene and protein), we could substantially increase predictive accuracy. However, multidimensionality engenders significant computational complexities and challenges. Whereas in single dimension analysis the number of considered variables in our case is relatively limited at 188, multidimensional analysis of two and three variables yields 17,578 and 1,089,836 combinations, respectively. Controlling for type 1 errors using cross-validation becomes critically important as the number of variables rises. For this reason, after excluding 17 patients due to incomplete data, we randomly assigned the remaining 250 patients to either training or testing set (Tab. 1). As a first step, we randomly chose either two or three variables from all the microRNAs, genes and proteins that we considered. After sufficient dimension reduction, variables were combined into a new diagnostic score, which included all the information of the parental factors. Computation clearly demonstrated that by increasing the amount of data from different dimensions, the calculated AUCs increased in the training set (Fig. 5A). After computation of all the 1,089,836 multidimensional predictors, we selected the combinations with the highest ranking of AUCs. We then added one additional biomarker at a time, a microRNA, gene or protein, into the existing combinations while considering all possible combinations, and calculated the AUCs again in the training set (Fig. 5B). This process was repeated until AUCs reached a maximum and failed to increase significantly by adding additional predictors into the existing combinations. These maximums were reached when number of variables inside each combination reached 10 in the training set (Fig. 5B). Thereafter, we analyzed the top combinations in the testing set and we found 15 multidimensional biomarkers (MB) which showed AUC values >0.83 in the training and testing set (composition is reported in Table 6, 7 and 8) supporting the notion that multidimensional biomarkers are more accurate than any individual single dimension predictor. From this list, we selected the 4 most accurate multidimensional biomarkers (MB1 to MB4), each with AUCs of approximately 0.9 in both training and test sets (Fig. 6A). Their composition is graphically depicted in Fig. 6B. These biomarkers were also outstanding predictors of outcome in Kaplan-Meier analysis (Fig. 6C).

**Table 6:** AUC analysis for the top 15 multidimensional biomarkers in the training and testing set.

AUC Combinations	MB	Training	Test
DICER1+IGFBP3+Mir−29a+HGF_Index +ADAMTS5+OSBPL3_Cyto_AQUA+ETS2+EPHA3+SHMT2+ESR1	1	0.91059	0.89881
ADAMTS5+OSBPL3_Cyto_AQUA+HGF_Index+ESR2+ANGPT2+HGF_Nuclear_AQUA+ARNT2+IGFBP3+Mir−29a+HGF_Cyto_AQUA	2	0.921007	0.871599
CD109+IGFBP3+Let−7c+CISH+KLF7+CDX1+MITF+ADAMTS5+ANO1+ANTXR2	3	0.914251	0.861305
HOXB7+IGFBP3+Mir−141+HGF_Index+ESR1+ANO1+PLK2+OSBPL3_AQUA+COX7A2L+IGFBP3_Stroma_AQUA	4	0.865103	0.899916
HOXB7+IGFBP3+MiR−320+HGF_Index+DROSHA+KLF6+Mir−200a+FGFR1+TM4SF1+Mir−200b	5	0.916084	0.847826
IGFBP3+Mir−29a+PNRC1+HGF_Index+CFTR+SRC+KLF5+KIF11+ANO1+CAV2	6	0.903652	0.855797
EZR+Mir−17+HGF_Index+ANO1+IGFBP3_AQUA+OSBPL3_AQUA+Mir−200b+PIM1+ANO1_AQUA+PPP2CA	7	0.827957	0.930193
EZR+Mir−200a+HGF_Index+IGFBP3+Mir−17+ESR1+Mir−92a+ERBB2+DUSP10+PLK2	8	0.868687	0.881884
DICER1+IGFBP3+MiR−193a−5p+CDX1+DROSHA+ANTXR2+HGF_Index+KIF11+OSBPL3_Cyto_AQUA+PLK2	9	0.875868	0.872449
RAI14+HGF_Index+STC1+MiR−18a+COX7A2L+SOX2+SMCR7L+NAT2+MCL1+ANTXR2	10	0.845377	0.88913
HOXB7+IGFBP3+Mir−141+HGF_Index+ESR1+ANO1+PLK2+OSBPL3_AQUA+COX7A2L	11	0.844618	0.885606
IGFBP3+Mir−29a+PNRC1+HGF_Index+CFTR+SRC+KLF5+KIF11+ANO1	12	0.885781	0.843478
HOXB7+IGFBP3+MiR−320+HGF_Index+DROSHA+KLF6+Mir−200a+FGFR1	13	0.862471	0.845652
DICER1+IGFBP3+Let−7g+CDX1+ANTXR2+ADM+DROSHA+KLF6+TUBB3_Cyto_AQUA+HGF_Cyto_AQUA	14	0.86748	0.837662
EZR+Mir17+HGF_Index+ANO1+IGFBP3_AQUA+OSBPL3_AQUA+Mir−200b+PIM1+ANO1_AQUA	15	0.839687	0.849453

**Table 7:** KM and Cox information in training set for all the top multidimensional biomarkers.

MB	P-value from KM	P-value from Cox	Range risk ratio	Range risk ratio lower limit	Range risk ratio upper limit
1	2.00E-07	1.51E-06	653.0181	46.55881	9159.011
2	1.28E-07	3.80E-06	258.9312	24.54175	2731.889
3	1.48E-09	7.80E-07	327.9436	32.9403	3264.907
4	6.92E-08	1.15E-05	331.7012	24.79851	4436.787
5	1.07E-09	1.24E-05	40807.52	349.2252	4768423
6	9.46E-08	2.72E-06	112.5715	15.63995	810.2548
7	1.09E-06	0.000413	195.6531	10.46789	3656.91
8	1.75E-05	2.97E-05	266.2441	19.35674	3662.079
9	9.17E-06	0.000101	192.0735	13.57356	2717.948
10	1.67E-06	6.71E-05	12784.75	122.3606	1335805
11	1.81E-05	5.65E-06	102.7509	13.90232	759.4232
12	7.40E-08	3.94E-06	560.1007	38.10062	8233.798
13	3.96E-08	6.38E-05	32337.23	199.1264	5251420
14	2.69E-05	0.000165	402.8112	17.78357	9123.973
15	0.000179	0.000116	89.50607	9.105603	879.8249

**Table 8:** KM and Cox information in testing set for all the top multidimensional biomarkers.

MB	P-value from KM	P-value from Cox	Range risk ratio	Range risk ratio lower limit	Range risk ratio upper limit
1	1.08E-07	0.000175	149.4211	10.9347	2041.818
2	8.10E-07	0.000655	135.4344	8.046889	2279.45
3	1.00E-05	2.96E-07	134.664	20.66007	877.7509
4	1.02E-06	0.000173	174.0681	11.78516	2571.004
5	0.000107	0.000499	64.97979	6.196342	681.43
6	1.51E-06	0.000702	75.67874	6.197425	924.1375
7	1.21E-09	6.37E-07	301.1185	31.85225	2846.654
8	2.45E-08	7.39E-06	1150.195	52.76299	25073.42
9	1.17E-06	2.19E-05	165.1841	15.62799	1745.956
10	0.003986	0.008584	33.05786	2.43376	449.0262
11	0.000976	0.000481	171.8073	9.553675	3089.673
12	0.000404	0.003664	16.68279	2.499333	111.3559
13	0.00047	0.002143	657810	126.8559	3.41E+09
14	2.47E-05	2.90E-05	143.1154	13.97101	1466.037
15	0.000149	4.68E-06	647.3492	40.53591	10338.02

## Discussion

CRC cancer remains among the deadliest malignancies. For clinical management, particularly in stage II and III disease, multiple therapeutic options are now available. As observed also in our clinical study the outcome is mostly driven by clinical stage at diagnosis with stage IV patients presenting a severe prognosis. However, even in patients with earlier stages the outcome is not only favorable with a significant relapse rate. Discoveries of effective biomarkers that can guide therapeutic decisions are ambitiously sought in the hopes of achieving the best possible outcomes, minimizing not necessary and toxic procedures. A host of studies have been conducted toward this end [2], [16], [17]. The ideal biomarker to drive clinical treatments should be significant in multivariate analysis while having robust predictive accuracy with few false positive and false negative results. Some limited successes have been obtained with regard to the selection of specific therapeutic regimens according to toxicity and efficacy [2]. However, most of these promising individual biomarkers have fallen short in clinical trials [18]. More complex biomarkers have been created, albeit in a single dimension. One 12-gene panel was effective in predicting risk of recurrence and response to treatment in a large clinical study of 1436 patients of stage II and III CRC patients [19]. However, later validation studies did not reproduce the same results [20], since the Achilles' heel of this technology remains the lack of accuracy in independent validation studies. In our study we intend dimension as the nature of the variable, being microRNA, a gene or a protein. We believe that the lack of accuracy should be dependent at least in part from the fact that the 12-gene signature was obtained only in the gene dimension, thus downsizing the possible role that other factors such as microRNA and protein may have in the predictive capability of genes. This past experience prompted us to revisit the way predictive biomarkers are built. Cancer aggressiveness is a complex trait in most of the cases. It is like a multifactorial equation. To make the pattern more complex, such multiple factors are coming from different dimensions such as genes, proteins, DNA sequences and different subset of cells (cancer and stromal). Our idea was centering the prediction on an integrated method of analysis including more dimensions and more factors at the same time. We believe that only an integrated approach can get closer to the solution of a multifactorial equation. The results we present in this study support our hypothesis. In our cohort of CRC patients, we first analyzed a large panel of possible individual predictors coming from each of three single dimensions (microRNA, gene or protein). We were indeed able to identify statistically significant predictors of outcome as determined by multivariate Cox analysis and Kaplan-Meier method. Some of these predictors have not been extensively investigated in CRC to date. As an example, expression of ANO1 (anoctamine 1) was found to be statistically significant at the gene and protein level, re-enforcing recent data coming from analysis of the dataset GSE14333 [15]. However, AUC of ANO1 in our analysis was not greater than 0.65, meaning that as a driver of clinical decisions, ANO1 would misclassify a consistent number of patients. Thus, statistical significance does not necessarily translate into clinical utility. Failure to recognize this fact can account for much of the disappointment with individual biomarkers derived from a single dimension [4], [20].

Not satisfied with AUCs below 0.7 and in the hopes of developing more robust predictors, we sought to combine our data in novel ways. In this manuscript, we provide details of a multidimensional platform which combines nanofluidic technology with quantitative fluorescent immunohistochemistry to create biomarkers with AUCs approaching and even exceeding 0.9. While the number of variables that need to be analyzed is immense, this potent toolset can collect multidimensional data at a reasonable reagent cost for FFPE samples ($0.20 for gene/microRNA analysis, $0.85 per protein).

Beyond predicting clinical outcome, our assay can highlight molecular drivers of aggressiveness. For example, IGFBP3 appears in all of the four top multidimensional biomarkers. This antigen is well known to researchers in CRC, although conflicting data are present in the literature regarding its effects [21]. At the gene expression level in both GSE14333 and our data set, high expression of IGFBP3 was related to poor outcome. This is in keeping with other previous studies [21], [22], [23]. The weight of evidence surely implicates this gene as a prominent driver of CRC cancer aggressiveness despite its being at odds with older studies connecting IGFBP3 expression to an anti-proliferative effect on the growth of colon cancer cells (reviewed in [24]).

Only two variables were present in 3 out of the 4 top multidimensional biomarkers: ADAMTS5 and HGF index. ADAMTS5 is a member of the ADAMTS (a disintegrin and metalloproteinase with thrombospondin motifs) protein family. The enzyme encoded by this gene contains two C-terminal TS motifs and functions as aggrecanase to cleave aggrecan, a major proteoglycan of cartilage [25]. As single factor, in the dataset GSE14333, high expression was associated with poor outcome in multiple probes. However, in our analysis, this factor did not show a significant trend in multivariate analysis as single element. Literature on ADAMTS5 in CRC cancer is extremely limited with only one study reporting this gene as one of the most hypermethylated in tumor as compared with the surrounding normal colonic mucosa [26]. The other variable, HGF (hepatocyte growth factor) index, represents a pathway that is known to be activated in aggressive CRC. HGF has been extensively investigated as a potential new target (reviewed in [27]). Although HGF expression in immunoperoxidase staining appears with a clear cytoplasmic pattern in CRC cancer cells [28], our immunofluorescence assay demonstrated a nuclear pattern that was of clinical significance. A similar nuclear localization of the receptor of HGF c-Met has been reported in breast cancer cells, where such overexpression was related to increased metastatic potential and aggressive disease [29].

In summary, CRC cancer aggressiveness is a complex trait that cannot be predicted with suitable accuracy by the use of an individual, single dimensional factor (microRNA, gene or protein). In contrast, a multidimensional integrated approach which utilizes data from microRNA, gene and protein analysis can generate accurate predictors of biological behavior, foster better clinical management of CRC, and shine a spotlight on molecules and molecular pathways which are associated with and potentially the cause of poor outcome.

## Supporting Information

Table S1 **List of antibodies, suppliers and final concentration used.**
(DOCX)
