# Supplementary material for: Integrated Multidimensional Analysis Is Required for Accurate Prognostic Biomarkers in Colorectal Cancer
Source: PLoS One. 2014 Jul 2;9(7):e101065. doi: 10.1371/journal.pone.0101065 (PMC4079703; doi:10.1371/journal.pone.0101065)
Supplement: Table S1 — List of antibodies, suppliers and final concentration used. (DOCX) [file pone.0101065.s001.docx]

Supplementary Table S1: List of antibodies, suppliers and concentrations used

| **Antigen** | **Isotype** | **Suppliers** | **Dilution** |
| --- | --- | --- | --- |
|  |  |  |  |
| ANO1 | rabbit polyclonal | Atlas Antibodies, #HPA032148 | (1:100) |
| ARNT2 | rabbit polyclonal | Santa Cruz, clone M-165, #sc-5581 | (1:200) |
| GLI3 | rabbit polyclonal | Sigma, #HPA005534 | (1:200) |
| HGF | mouse IgG_1_ | Santa Cruz, clone H-10, #sc-374422 | (1:100) |
| IGFBP3 | rabbit polyclonal | Santa Cruz, clone H-98, #sc-9028 | (1:100) |
| OSBPL3 | rabbit polyclonal | Sigma, #HPA005534 | (1:50) |
| PP2A | rabbit polyclonal | Abcam, # ab137849 | (1:200) |
| TUBB3 | mouse IgG_1_ | Santa Cruz, clone TUJ-1, #sc-58888 | (1:500) |
| Cytokeratin | mouse/AE1/AE3 | DAKO, #M3515012 | (1:50) |
| Cytokeratin | rabbit polyclonal | DAKO, #Z0622 | (1:50) |
| Vimentin | chicken | Millipore, #AB5733 | (1:200) |
|  |  |  |  |
| **Secondary Antibody** |  | **Company** | **Dilution** |
| Envision anti-rabbit | H+L | DAKO, #K4003 | Neat |
| Envision anti-mouse | H+L | DAKO, #K4001 | Neat |
| Alexa555-conjugated goat anti-mouse | IgG1 | Invitrogen, #A21422 | (1:100) |
| Alexa555-conjugated goat anti-rabbit | IgG (H+L) | Invitrogen, #A21428 | (1:100) |
| Alexa488-conjugated goat anti-chicken | IgG (H+L) | Invitrogen, #A11039 | (1:100) |
